# Supplementary material for: NMR-Based Metabolomics: A New Paradigm to Unravel Defense-Related Metabolites in Insect-Resistant Cotton Variety through Different Multivariate Data Analysis Approaches
Source: Molecules. 2023 Feb 13;28(4):1763. doi: 10.3390/molecules28041763 (PMC9966674; doi:10.3390/molecules28041763)
Supplement: Supplementary file 1 [file molecules-28-01763-s001.zip › molecules-2032775-supplementary.pdf]

## Supplementary Material

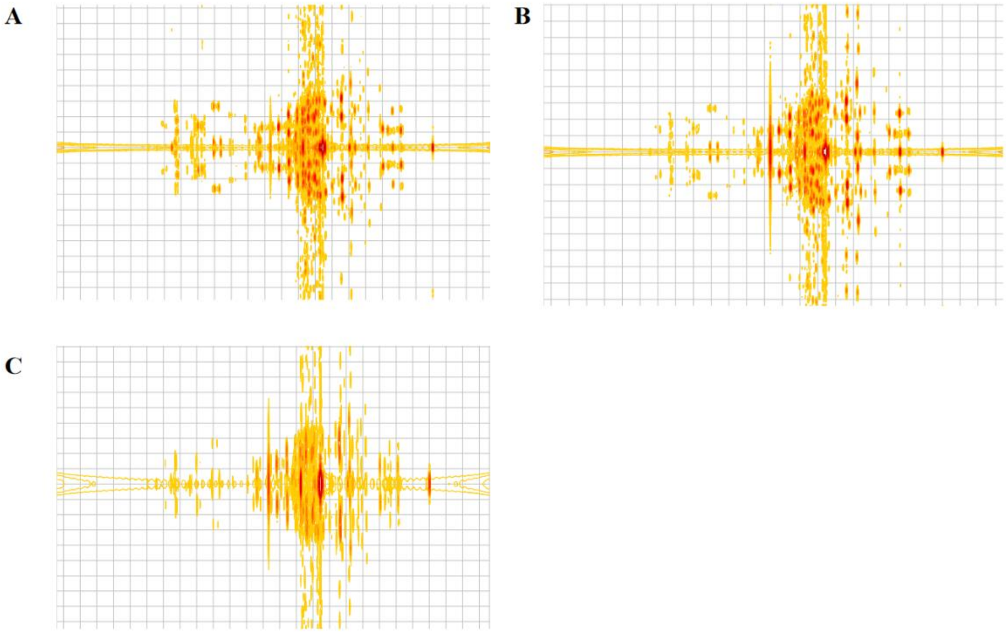


**Supplementary Figure S1 (A) 2D J-resolve of FH-142; (B) 2D J-resolve of 496; (C) 2D J-resolve of MAC**

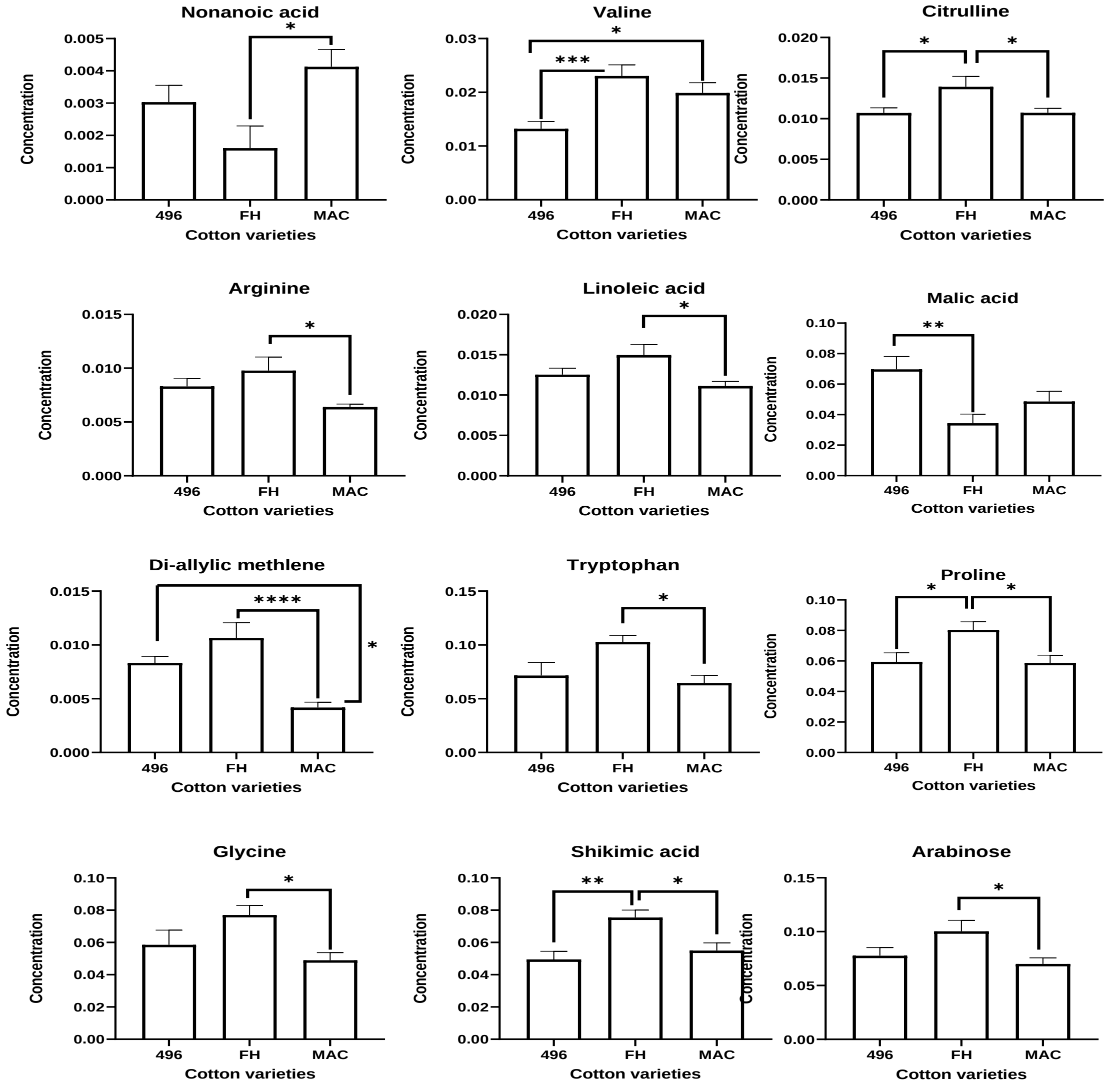


**Figure S2 (A) Relative quantification of metabolites**

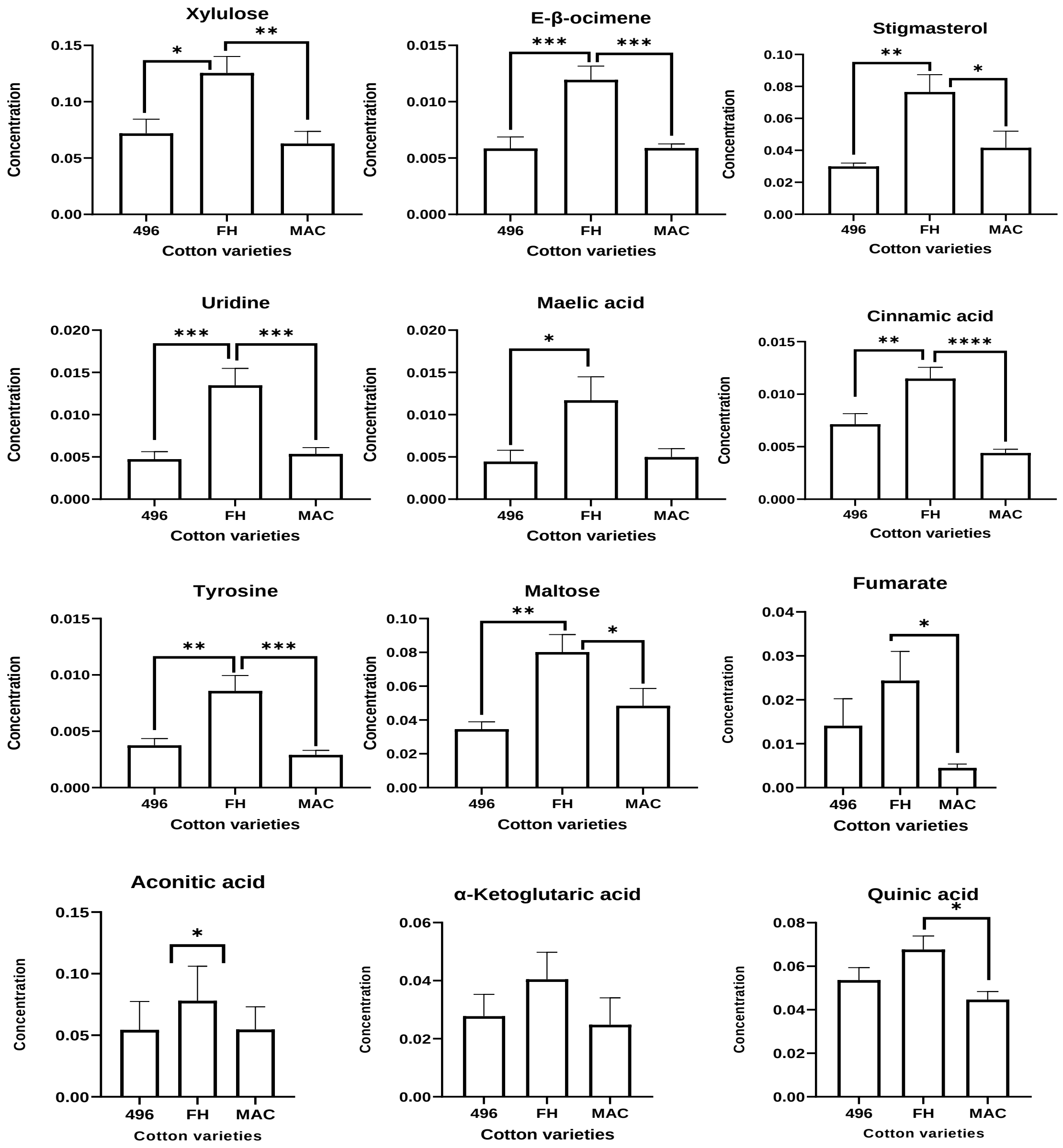


**Figure S2 (B) Relative quantification of metabolites**

Supplementary Table S1 Relative quantification of metabolites in different cotton varieties

| Sr. No. | Metabolites | Concentration of metabolites ± SEM | | | Probability | | |
|---|---|---|---|---|---|---|---|
| | | 496 | FH | MAC | 496×FH | 496×MAC | FH × MAC |
| **1** | Nonanoic acid | 0.0030 ± 0.0005 | 0.0016 ± 0.0006 | 0.0041 ± 0.0005 | ns | Ns | * |
| **2** | Valine | 0.0132 ± 0.0013 | 0.0230 ± 0.0020 | 0.0198 ± 0.0019 | *** | * | ns |
| **3** | Citrulline | 0.0107 ± 0.0006 | 0.0139 ± 0.0012 | 0.0107 ± 0.0005 | * | Ns | * |
| **4** | Arginine | 0.0083 ± 0.0007 | 0.0097 ± 0.0012 | 0.0063 ± 0.0002 | ns | Ns | * |
| **5** | Malic acid | 0.0697 ± 0.0083 | 0.0343 ± 0.0060 | 0.0486 ± 0.0066 | ** | Ns | ns |
| **6** | Di-allylic methylene | 0.0083 ± 0.0006 | 0.0107 ± 0.0014 | 0.0041 ± 0.0005 | ns | ** | **** |
| **7** | Asparagine | 0.0053 ± 0.0005 | 0.0052 ± 0.0013 | 0.0021 ± 0.0005 | ns | * | * |
| **8** | Tryptophan | 0.0716 ± 0.0123 | 0.103 ± 0.0061 | 0.0646 ± 0.0071 | ns | Ns | * |
| **9** | Proline | 0.0594± 0.0058 | 0.0804 ± 0.0052 | 0.0587 ± 0.0050 | * | Ns | * |
| **10** | Glycine | 0.0585 ± 0.0090 | 0.0771 ± 0.0059 | 0.0489 ± 0.0047 | ns | Ns | * |
| **11** | Shikimic acid | 0.0494 ± 0.0051 | 0.0755 ± 0.0045 | 0.0550 ± 0.0047 | ** | Ns | * |

| | | | | | | | |
|---|---|---|---|---|---|---|---|
| **12** | Arabinose | 0.0777 ± 0.0074 | 0.100 ± 0.0102 | 0.0701 ± 0.0055 | ns | Ns | * |
| **13** | Xylulose | 0.0719 ± 0.0126 | 0.126 ± 0.0146 | 0.0629 ± 0.0108 | * | Ns | ** |
| **14** | E-β-ocimene | 0.0058 ± 0.0010 | 0.0119 ± 0.0012 | 0.0058 ± 0.0003 | *** | Ns | *** |
| **15** | Stigmasterol | 0.0299 ± 0.0020 | 0.0765 ± 0.0109 | 0.0416 ± 0.0104 | ** | Ns | * |
| **16** | Maltose | 0.0346 ± 0.0043 | 0.0802 ± 0.0104 | 0.0484 ± 0.0102 | ** | Ns | * |
| **17** | Uridine | 0.0047 ± 0.0008 | 0.0135 ± 0.0020 | 0.0053 ± 0.0007 | *** | Ns | *** |
| **18** | Maelic acid | 0.0044 ± 0.0013 | 0.0117± 0.0027 | 0.0050 ± 0.0009 | * | Ns | ns |
| **19** | Cinnamic acid | 0.0071 ± 0.0010 | 0.0115 ± 0.0010 | 0.0044 ± 0.0003 | ** | ns | **** |
| **20** | Tyrosine | 0.0037 ± 0.0005 | 0.0085 ± 0.0013 | 0.0029 ± 0.0003 | ** | Ns | *** |
| **21** | Fumarate | 0.0141± 0.0061 | 0.0243± 0.066 | 0.0045± 0.0008 | ns | Ns | * |
| **22** | Aconitic acid | 0.0541± 0.0066 | 0.0780± 0.0080 | 0.0546± 0.0053 | * | Ns | ns |
| **23** | α-Ketoglutaric acid | 0.0277± 0.0075 | 0.0404± 0.0093 | 0.0248± 0.0092 | ns | Ns | ns |
| **24** | Succinic acid | 0.0176± 0.0093 | 0.0315± 0.0172 | 0.0134± 0.0048 | ns | Ns | ns |
| **25** | Quinic acid | 0.0536± 0.0057 | 0.0677± 0.0061 | 0.0446± 0.0036 | ns | Ns | * |
| **26** | Ferulic acid | 0.0047± 0.0008 | 0.0090± 0.001 | 0.0045± 0.0004 | ** | Ns | *** |
